# Global prevalence of eligibility for biologic therapy in ATS/ERS-defined severe asthma: A systematic review^☆^

**DOI:** 10.1016/j.waojou.2025.101155

**Published:** 2025-12-11

**Authors:** Freda Yang, Justin D. Salciccioli, Rhea E. Patel, Marcus McClean, Chloe I. Bloom

**Affiliations:** a National Heart and Lung Institute, Imperial College London, London, United Kingdom; b Royal Brompton Hospital, Guy's and St Thomas' NHS Foundation Trust, London, United Kingdom; c Division of Pulmonary and Critical Care Medicine, Brigham and Women's Hospital, Harvard Medical School, Boston, United States; d Imperial College School of Medicine, Imperial College London, London, United Kingdom; e Imperial College Healthcare NHS Trust, London, United Kingdom

**Keywords:** Asthma, Biologics, Eligibility, Prevalence, Type-2 inflammation

## Abstract

**Background:**

Biologic therapies improve outcomes in severe asthma, but eligibility criteria vary globally, influencing the proportion of patients who qualify. We systematically reviewed studies to estimate the global prevalence of biologic eligibility in patients aged ≥12 years with American Thoracic Society / European Respiratory Society (ATS/ERS)-defined severe asthma and the proportion eligible for each biologic.

**Methods:**

Following PRISMA guidelines (PROSPERO CRD42023393897), we searched MEDLINE, EMBASE, Web of Science, and ClinicalTrials.gov for studies published between 2000 and 2025 that reported the proportion of biologic-naïve, severe asthma patients eligible for omalizumab, mepolizumab, benralizumab, reslizumab, dupilumab, or tezepelumab. Two reviewers independently screened studies, extracted data on eligibility proportions and criteria, and assessed quality using the AXIS tool.

**Results:**

Ten observational studies, including 3500 patients with ATS/ERS-defined severe asthma, met the inclusion criteria. Across all studies, 1770 patients (51%) were eligible for at least 1 biologic, though estimates ranged widely from 24% to 91%, largely reflecting differences in national eligibility criteria. Omalizumab eligibility was reported in 8 studies (16%, range 6%–66%), mepolizumab in 9 studies (27%, 19%–78%), benralizumab in 6 studies (25%, 19%–53%), reslizumab in 6 studies (17%, 6%–41%), and dupilumab in 2 studies (41%, 37%–75%). No study assessed tezepelumab. Overall, the lowest eligibility (24%) was reported in the European IDEAL cohort due to stringent exacerbation and biomarker criteria, whereas the highest (91%) was observed in a Canadian single-centre cohort using less restrictive national regulatory criteria.

**Conclusion:**

Globally, approximately 51% of adults with severe asthma are eligible for biologic therapy, excluding tezepelumab. Among available biologics, eligibility is generally higher for anti-IL5/IL5Rα therapies than for anti-IgE, and appears highest for anti-IL4Rα, although data for the latter remain limited.

## Introduction

Asthma affects approximately 262 million people worldwide,^1^ and approximately 5–10% of these individuals have severe asthma.^2^ Although this group represents a small subset of all asthma patients, they disproportionately contribute to healthcare costs, morbidity, and mortality.^3^^,^^4^ Severe asthma is associated with frequent exacerbations, increased hospitalizations, and reduced quality of life. Monoclonal antibodies targeting Type-2 (T2) inflammation, commonly known as biologic therapies, are effective treatments for moderate-to-severe asthma. They reduce exacerbations, lower systemic corticosteroid exposure, and improve disease-related morbidity and quality of life.^5^

Currently, 6 biologic therapies are approved by the United States (US) Food and Drug Administration (FDA) and the European Medicines Agency (EMA) for the treatment of asthma: omalizumab, mepolizumab, benralizumab, reslizumab, dupilumab, and tezepelumab.^5^ Omalizumab (anti-IgE) is effective in the allergic asthma phenotype, while mepolizumab and reslizumab (anti-IL5) and benralizumab (anti-IL5Rα) target eosinophilic inflammation, which is strongly associated with frequent exacerbations. Dupilumab (anti-IL4Rα) blocks IL-4 and IL-13, key mediators of T2 inflammation.^6^ Tezepelumab, the latest biologic approved for use, targets thymic stromal lymphopoietin (TSLP) and is licensed for use irrespective of T2 biomarker status.^5^

Eligibility criteria for biologics as defined by the EMA and FDA are largely based on the inclusion criteria of randomized controlled trials (RCTs) demonstrating treatment efficacy.^5^ However, real-world access varies widely, as many countries have stricter national prescribing criteria.^7^ For example, the International Severe Asthma Registry (ISAR) study examined differences across 28 countries and found that most required a diagnosis of severe asthma and some evidence of T2 inflammation.^8^ This typically includes allergic sensitization for omalizumab, blood eosinophilia for anti-IL5/IL5Rα agents (with varying thresholds), or elevated FeNO for anti-IL4Rα therapy.^5^

These variations in national criteria are largely driven by cost-effectiveness considerations and regulatory differences across healthcare systems.^9^ Previous studies have assessed the prevalence of biologic eligibility in severe asthma within individual countries, but such variability makes it difficult to estimate the global burden.^10^, ^11^, ^12^, ^13^, ^14^, ^15^, ^16^, ^17^ Understanding the global prevalence of biologic-eligible patients is important for highlighting international disparities in access, informing policy and economic planning for biologic therapy, and estimating the potential health and societal impact of expanding or rationalizing biologic use. To address this gap, we conducted a systematic review to determine the prevalence of biologic eligibility among patients with severe asthma worldwide.

## Methods

### Registration and search

This systematic review followed the PRISMA for systematic review protocol (PRISMA-P). The review protocol was registered on the International Prospective Register of Systematic Review (PROSPERO registration number CRD42023393897). Two reviewers, [Author 1] and [Author 2], independently searched MEDLINE, EMBASE, Web of Science, and ClinicalTrials.gov. The search strategy combined the keywords “asthma”, “biologic” and “eligib∗”, with variations of each term. Variations of “biologic” included “monoclonal”, “mAB”, “omalizumab”, “mepolizumab”, “benralizumab”, “reslizumab”, “dupilumab”, and “tezepelumab”. Variations of “eligib∗” included “suitab∗” and “qualify”.

Study selection was based on predefined eligibility criteria for study design, patient population, publication year, and pre-specified exclusion criteria (Supplement E1). Discrepancies in study selection were resolved by consensus between the 2 reviewers, with a third reviewer [Author 5] adjudicating if disagreements persisted.

### Data selection

We included studies of patients aged ≥12 years with a diagnosis of American Thoracic Society / European Respiratory Society (ATS/ERS)-defined severe asthma; “requiring treatment with high dose inhaled corticosteroids plus a second controller and/or systemic corticosteroids to prevent asthma from becoming uncontrolled or that remains uncontrolled despite this therapy”.^2^ Publications in English between January 2000 and April 2025 were eligible. The primary inclusion criterion was reporting the number, percentage or proportion of biologic-naïve severe asthma patients eligible for 1 of the 6 currently approved biologics (omalizumab, mepolizumab, benralizumab, reslizumab, dupilumab, and tezepelumab). There were no restrictions by publication type; conference abstracts, correspondences and original articles were all considered. Studies were excluded if they: reported only trial design, used duplicate patient cohorts, or included patients already receiving biologics. Studies conducted in mild, moderate, difficult-to-treat, or undefined asthma populations were reviewed and included only as a supplementary analyses.

### Data extraction

Two reviewers [Author 1 and Author 2] independently extracted data using a predefined template. Extracted variables included:

• Study characteristics (publication type, study design, population)
• Proportion of patients eligible for any biologic
• Proportions eligible for specific biologics (if reported)
• Eligibility criteria used to define biologic candidates

For conference abstracts, additional data were extracted from subsequent conference posters where available. Disagreements in data extraction or quality assessment were resolved by consensus, with a third reviewer [Author 4 and Author 5] adjudicating if needed. We assessed study quality using the Appraisal Tool for Cross-Sectional Studies (AXIS) and computed simple descriptive statistics,^18^ including counts (percentages) and medians (IQR), to summarize the findings.

## Results

### Characteristics of included studies

**Fig. 1:**
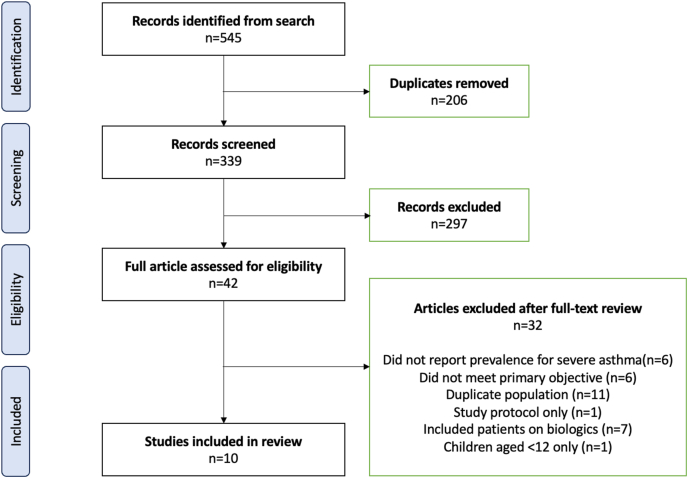
PRISMA flow diagram of the literature search

The study selection process is summarised in the PRISMA flow diagram (Fig. 1). Ten studies were included, comprising 6 original articles,^12^, ^13^, ^14^^,^^16^^,^^17^^,^^19^ 2 research letters^10^^,^^15^ and 2 conference abstracts.^11^^,^^20^ All publications were from 2009 to 2025 and included data spanning 1999 to 2020 (Table 2). All studies were observational; 9 were cross-sectional and 1 was a cohort study (Table 1).

**Table 1:** Proportion of patients aged ≥12 years with ATS/ERS-defined severe asthma eligible for biologic therapy, and the proportion eligible for each individual biologic.

First author and Year	Study design	Age (years)	Country	Severe asthma (n)	Percentage of severe asthma patients eligible for biologics	Percentage of severe asthma patients eligible for each biologic agent				
						Omalizumab	Mepolizumab	Benralizumab	Reslizumab	Dupilumab
**Albers 2018**	Cross-sectional	≥12	France, UK, Germany (Group 1) Australia, USA, Canada (Group 2)	502	24.1% (Group 1) 34.7% (Group 2)	7.4% (Group 1) 21.3% (Group 2)	20.1%	✕	5.6%	✕
**Deng 2023**	Cross-sectional	≥18	China	452	38.5%	11.3%	19.3%	19.3%	19.3%	36.7%
**Lee 2019**	Cross-sectional	≥18	Korea	809	39.7%	6.3%	20.1%	✕	✕	✕
**Comberiati 2019**^a^	Cross-sectional	≥6	USA	157	38.0% (Eos ≥300/μL) 55.1% (Eos ≥150/μL)	✕	38.0% (Eos ≥300) 55.1% (Eos ≥150)	✕	✕	✕
**Marques Mello 2021**^b^	Cohort study	≥18	Brazil	172	59.9%	34.9%	35.5%	18.6%	16.9%	✕
**Kanniess 2021**^c^	Cross-sectional	≥18	Europe (12 countries)	1025	62%	✓	✓	✓	✓	✓
**Lee 2018**^d^	Cross-sectional	≥18	Australia	59	64.4%	43.5%	31.9%	31.9%	31.9%	✕
**Akenroye 2020**^e^	Cross-sectional	≥6	USA	59	83.9%	41%	66.9%	32.3%	19.5%	75.1%
**Menzella 2020**^f^	Cross-sectional	≥12	Italy	137	91%	7.3%	19.7%	19.0%	✕	✕
**Jeimy 2018**	Cross-sectional	≥12	Canada (Ontario)	128	91.4%	66%	78%	53%	41%	✕

✓ Included but individual percentages were unavailable.

✕ Not included.

a Comberiati (2019) included patients aged ≥6 years; only adults with severe asthma are reported here, as per review guidelines.

b Marques Mello (2021) used a modified ATS/ERS severe asthma definition; treatment with high doses of inhaled corticosteroid, >1000 mcg of fluticasone or equivalent, plus the use of an additional controller, at least 6 months.

c The 12 countries included Bulgaria, Czechia, France, Germany, Greece, Hungary, Italy, Netherlands, Poland, Romania, Slovenia, Spain. The percentage of patients eligible for each biologic was not reported.

d Lee (2018) included patients with difficult-to-treat asthma; however, only those with severe asthma are shown in the table. The percentage of patients eligible for each anti-IL5 biologic was not reported separately; instead, the overall percentage of biologic-eligible patients was provided.

e Akenroye (2020) included patients aged ≥6 years; only adults with severe asthma are reported here, as per review guidelines.

f Menzella (2020) used the ATS/ERS severe asthma definition plus the GINA 2018 classification, the latter of which was defined as “asthma that is uncontrolled despite adherence to with maximal optimised therapy and treatment of contributory factors, or that worsens when high dose treatment is decreased”.

**Table 2:** Characteristics of include studies.

First Author	Publication year	Article Type	Journal or Conference	Data source	Study start date	Study end date
**Albers FC**	2018	Original article	Journal of asthma	Severe asthma patients recruited from primary and specialist care	2014	2015
**Deng**	2023	Research letter	Chinese Medical Journal	Chinese Biomarkers for the Prediction of Respiratory Disease Outcomes (C-BIOPRED), recruited severe asthma patients from 33 university hospital in 15 provinces in China.	Unavailable	Unavailable
**Lee JH**	2019	Conference abstract	EAACI	Cohort of Reality and Evolution of Adult Asthma in Korea (COREA)	Unavailable	Unavailable
**Comberiati P**	2019	Original article	JACI in practice	Single centre, National Jewish Health	2010	2013
**Marques Mello L**	2021	Original article	Journal of asthma	Program for Control of asthma (ProAR) in Bahia	2013	2015
**Kanniess F**	2021	Conference abstract	ERS	Primary and secondary care in Europe	April 2018	July 2020
**Lee Joy**	2018	Original article	ERJ	Patients referred by respiratory or allergy speciality with difficult asthma	01/05/2015	31/12/2016
**Akenroye A**	2020	Research letter	JACI	National Health and Nutrition Examination Survey (NHANES)	2005	2012
**Menzella F**	2020	Original article	Pul Pharm & Therapy	Single-centre, patients referred to Pneumology unit of Azienda USL di Reggio Emilia / Scientific Institute for Research, Hospitalization and Healthcare (IRCCS) Italy, and enrolled in Severe Asthma Network Italy (SANI)	01/06/2017	30/06/2019
**Jeimy S**	2018	Original article	Allergy asthma and clinical immunology	Single centre, community allergy and immunology practice	Unavailable	Unavailable

Abbreviations: EAACI, European Academy of Allergy and Clinical Immunology; JACI, Journal of Allergy and Clinical Immunology; ERS, European Respiratory Society; ERJ, European Respiratory Journal.

Of the 3500 patients with severe asthma included in this review, the largest cohort came from RECOGNISE (NCT03629782),^21^ a multicentre European observational study of 1025 adults from 12 countries.^20^ This AstraZeneca-funded study recruited severe asthma patients from primary and secondary care to describe the proportion and characteristics of biologic-eligible patients.^21^ We used data from the 2021 European Respiratory Society Congress abstract, as most patients from subsequent country-specific publications were already represented in this publication.^20^ The remaining 2475 severe asthma patients in this review were from 9 other studies spanning across 5 continents, with 2 studies including patients from Europe,^16^^,^^19^ 4 from North America,^12^^,^^15^^,^^17^^,^^19^ 2 from Asia,^10^^,^^11^ 2 from Australia,^14^^,^^19^ and 1 from South America^13^ (Table 1). Three of the 10 studies were single centre studies,^12^^,^^16^^,^^17^ while others used registry data, national health surveys, or recruited patients from primary care or specialist centres (Table 2).

Studies varied in the number of biologics assessed. Tezepelumab eligibility was not evaluated in any study, and only 2 studies assessed all other 5 biologics.^10^^,^^15^ All but 1 study evaluated both omalizumab and mepolizumab eligibility,^12^ likely reflecting their longer availability. Benralizumab^10^^,^^13^^,^^13^, ^13^, ^14^, ^15^, ^16^, ^17^^,^^20^ and reslizumab^10^^,^^13^, ^14^, ^15^^,^^17^^,^^19^^,^^20^ were assessed in 7 studies each, while dupilumab was evaluated in only 2 studies^10^^,^^15^^,^^20^ (Table 1).

The overall risk of bias was low from a non-interventional perspective, but selection bias was high, as none of the included studies were based on national severe asthma registries with compulsory patient enrolment. Several studies had pharmaceutical involvement which can introduce sponsorship bias through choices in study framing and analysis; 4 studies included authors employed by pharmaceutical companies,^10^^,^^12^^,^^13^^,^^19^ and 5 studies were industry-funded by GSK or AstraZeneca.^10^^,^^13^^,^^14^^,^^19^^,^^20^ Reporting quality varied. Three of the 10 studies did not specify their start and end dates.^10^^,^^11^^,^^17^ Eight studies were peer-reviewed, while the remaining 2 were conference abstracts^11^^,^^20^ accepted at an international European meeting following a formal selection process. Internal validity was generally reasonable, with appropriate study designs, statistical analyses, clearly stated aims, acknowledgement of limitations, and justified conclusions. However, 3 studies^11^^,^^15^^,^^20^ (2 abstracts and 1 research letter) did not report an ethics approval statement for human research.

### The proportion of patients eligible for any biologic therapy

Across all studies, the proportion of severe asthma patients eligible for at least 1 biologic ranged widely from 24% to 91%, with an overall estimate of 1770 patients (51%) out of 3500 meeting eligibility criteria. The lowest proportion (24%) was reported in the European subgroup of the IDEAL study, which included patients from France, United Kingdom, and Germany.^19^ This low rate reflects the strict European criteria for omalizumab, but even in the Australia, United States of America, and Canada cohorts within IDEAL, only 35% of patients were eligible. In contrast, the highest proportion (91%) came from a single-centre Canadian study of patients attending an allergy and immunology clinic in Ontario.^17^ Both studies evaluated eligibility for omalizumab and anti-IL5/IL5Rα biologics, but IDEAL did not assess benralizumab (Table 1). Importantly, the differences in reported prevalence reflected variation in the national eligibility criteria: IDEAL required ≥1 or ≥2 exacerbations in the prior year for biologic eligibility,^19^ whereas the Canadian study^17^ followed national regulatory criteria, where benralizumab required ≥1 exacerbation, and other biologics required only biomarker evidence and uncontrolled disease without a strict exacerbation threshold (Supplement E2 and E3).

### Proportion of patients eligible for individual biologics

Nine studies reported the proportion of severe asthma patients eligible for individual biologics, encompassing a total of 2475 patients.^10^, ^11^, ^12^, ^13^, ^14^, ^15^, ^16^, ^17^^,^^19^ Omalizumab eligibility was assessed in 2318 patients across 8 studies,^10^^,^^11^^,^^13^, ^14^, ^15^, ^16^, ^17^^,^^19^ with 377 patients (16%) eligible, ranging from 6% to 66%. Mepolizumab eligibility was reported in all 9 studies,^10^, ^11^, ^12^, ^13^, ^14^, ^15^, ^16^, ^17^^,^^19^ with 657 of 2475 patients (27%) eligible, ranging from 19% to 78%. Benralizumab^10^^,^^13^, ^14^, ^15^, ^16^, ^17^ and reslizumab^10^^,^^13^, ^14^, ^15^^,^^17^^,^^19^ eligibility were each evaluated in 6 studies, with 251 of 1007 patients (25%, range 19%–53%) and 227 of 1372 patients (17%, range 6%–41%) eligible, respectively. Most studies reported eligibility for anti-IL5/IL5Rα biologics individually, although 2 grouped them together. Dupilumab eligibility was reported in only 2 studies,^10^^,^^15^ with 210 of 511 patients (41%, range 37%–75%) eligible (Table 1 and Supplement E5).

### Differences in eligibility criteria world-wide

Differences in global biologic eligibility criteria are beyond the scope of this review and have been previously described by Porsbjerg et al.^8^ However, we documented the eligibility criteria applied in each included study to help explain the variability in reported prevalence. These criteria are summarised by biologic class (anti-IgE, anti-IL5/IL5Rα, anti-IL4Rα) in Supplementary Tables E2 to E4. The most common additional requirement beyond a diagnosis of severe asthma was a recent history of exacerbations treated with systemic corticosteroids, typically ≥1 or ≥2 in the previous 12 months. This threshold was applied more consistently for IL5/IL5Rα and anti-IL4Rα eligibility than for anti-IgE.

In general, studies assessing omalizumab eligibility required a history of allergic asthma with sensitization to perennial allergens, demonstrated by specific serum IgE or positive skin prick testing, and patients also needed to fall within the omalizumab dosing range, determined by total IgE level and body weight. Biomarker evidence of T2 inflammation using raised blood eosinophil count for anti-IL5/IL5Rα eligibility, and/or FeNO for anti-IL4Rα eligibility. Among studies assessing the prevalence of potential anti-IL5/IL5Rα candidates, the minimum blood eosinophil threshold varied. Five studies allowed for ≥150 cells/μL at the baseline study visit,^12^^,^^13^^,^^16^^,^^17^^,^^19^ otherwise ≥300 cells/μL or ≥400 cells/μL were generally considered acceptable if captured in the past 12 months. Some studies adjusted the minimum blood eosinophil count requirement based on exacerbation frequency and maintenance oral corticosteroid use^17^ (Supplement E3).

### Non-severe asthma populations

We identified 8 studies that met our inclusion criteria but were conducted in asthma populations not specific to ATS/ERS-defined severe asthma.^14^^,^^15^^,^^22^, ^23^, ^24^, ^25^, ^26^, ^27^ Two of these studies also reported eligibility in severe asthma subgroups,^14^^,^^15^ which were included in our main analysis, while the overall cohorts represented non-severe or mixed-severity asthma. As expected, the prevalence of biologic-eligible patients was generally lower in these populations, ranging from 1.4% to 55%. The lowest rate (1.4%) was observed in a single-centre London study of 2473 primary care asthma patients.^24^ In contrast, the highest rate (55%) was reported in an Australian cohort of difficult-to-treat asthma patients referred to a respiratory and allergy clinic.^14^ Results are summarised in Supplement Table E6 to E11.

## Discussion

This systematic review shows that approximately half of patients with ATS/ERS-defined severe asthma worldwide meet eligibility criteria for at least 1 biologic therapy targeting IgE, IL-5/IL-5Rα, or IL-4Rα. Reported prevalence varied widely, driven by differences in national regulatory criteria and heterogeneity in study populations. However, a recent history of exacerbations treated with systemic corticosteroids, typically ≥1 or ≥2 in the previous 12 months, appear to be a consistent global requirement for biologic eligibility. Studies recruiting from specialist centres consistently reported higher eligibility, while no study to date has evaluated a nationally representative severe asthma cohort. Our findings therefore provide the most comprehensive global estimate of biologic eligibility in severe asthma to date, which can serve as a benchmark for future research, health-economic planning, and policy development.

Our findings underscore that both patient population characteristics and national eligibility criteria strongly influence biologic eligibility, the latter of which contribute substantially to international disparities in access. Across the 10 studies conducted worldwide, more patients were eligible for anti-IL5/IL5Rα therapy than for anti-IgE therapy. Eligibility appeared to be highest for anti-IL4Rα therapy, but this finding was based on limited data from only 2 studies that examined it. These patterns are clinically important for anticipating future demand, optimizing biologic prescribing pathways, and guiding healthcare resource allocation.

Our study focused on biologic-naïve patients to provide the most accurate estimate of eligibility in a true global severe asthma population. This approach avoids potential bias in biologic-treated cohorts, which may overestimate eligibility due to enrichment with patients referred for biologics, or underestimate it when biomarkers are suppressed by ongoing therapy. Reassuringly, our findings are broadly consistent with results from biologic-treated populations. In a UK registry of 2490 severe asthma patients receiving biologics, mepolizumab was most commonly prescribed (44.8%), followed by benralizumab, omalizumab, reslizumab, and dupilumab.^28^ Similarly, we found mepolizumab had the highest global eligibility (27%) among anti-IL5/IL5Rα and anti-IgE therapies. By contrast, dupilumab eligibility was higher globally but lowest in the United Kingdom, reflecting restrictive national criteria requiring prior ineligibility for or failure of anti-IL5/IL5Rα therapy.^29^ Overall, 51% of ATS/ERS-defined severe asthma patients in our review were eligible for at least 1 biologic, slightly lower than the 66% of patients receiving biologics in CHRONICLE, a large US registry of subspecialist-diagnosed severe asthma. This difference likely reflects broader access to biologics in the United States compared with other countries.^8^

We did not observe clear intercontinental differences in the prevalence of biologic eligible severe asthma patients among countries with access. In Europe and North America, eligibility varied widely by country. For example, an Italian study^16^ and a Canadian cohort^17^ each reported very high eligibility rates (>90%), whereas the European subgroup in the IDEAL study^19^ and a US single-centre study at National Jewish Health^12^ reported much lower rates (24% and 38%, respectively). Prevalence of biologic eligible patients appeared to be lower in Asian cohorts (38%–40%), although conclusions are limited by the small number of studies available.^10^^,^^11^

Our prevalence findings are broadly consistent with the ISAR study, which assessed global biologic accessibility using the Biologic Accessibility Score (BACS), where higher scores indicate easier access.^8^ Reslizumab had the lowest BACS across participating countries, consistent with our finding of the lowest prevalence for biologic eligible compared to other anti-IL5/IL5Rα therapies. In ISAR, dupilumab had the highest mean BACS (59), followed by omalizumab (57), mepolizumab (55), benralizumab (54), and reslizumab (51), reflecting national criteria for dupilumab which were generally as permissive as, or less restrictive than EMA licensing. Similarly, we observed the highest global prevalence of eligibility for dupilumab (41%). The main discrepancy arose with omalizumab where despite its high BACS in ISAR, we found the lowest eligibility prevalence. This likely reflects the influence of the 2 larger studies in our review, Albers et al^19^ and Lee et al,^11^ both of which reported very low omalizumab eligibility. Albers et al applied additional spirometry criteria in some countries, which likely contributed to lower rates, but Lee et al followed Korea Food and Drug Administration (KFDA) criteria in South Korea, which closely resemble standard licensing requirements and are not particularly restrictive.^30^ Alternatively, the higher proportion eligible for anti-IL5/5Rα than anti-IgE likely reflect an enrichment for eosinophilic asthma. Many included studies required recent exacerbations to qualify as “biologic-eligible”, and while two-thirds of frequent exacerbators are atopic (with perennial allergen sensitization),^31^ exacerbation-prone severe asthma is more closely associated with eosinophilic inflammation.^32^ Therefore, by focusing on biologic candidates with history of exacerbations, we may have enriched for eosinophilic asthma and consequently increased eligibility for anti-IL5/5Rα relative to anti-IgE.

We found that variation in national eligibility criteria is a key driver of differences in the prevalence of biologic eligibility and, consequently, patient access. Minimum exacerbation history and biomarker measurements are central to these criteria. Most studies assessed blood eosinophil counts within a 12-month window and used a minimum cut-off of 150 cells/μL to 400 cells/μL. Any change or relaxation of this timeframe, or of minimum eosinophil thresholds between 150 cells/μL and 400 cells/μL, would likely have a substantial impact on the proportion of patients deemed eligible. Although our review could not fully disentangle these effects, they are critical to understanding reported prevalence.

A key strength of our study is the use of a rigorous, PRISMA-P–compliant methodology, including independent reviewers with an adjudicator, and systematic assessment of study quality and risk of bias using the AXIS tool. We carefully reviewed overlapping publications to avoid duplicate patient populations, ensuring that only unique cohorts were included. The study population was largely homogenous in that most studies used the ATS/ERS definition of severe asthma; 1 study used a modified, more stringent version that required a prior exacerbation. Of note, some patients with ATS/ERS-defined severe asthma may have qualified for biologics based on uncontrolled symptoms if prior exacerbation history was not a requirement.

Several limitations should be considered when interpreting our findings. First, 2 included studies were available only as conference abstracts, which provided limited methodological details and may be of lower quality. They were included due to the scarcity of data meeting our objectives. Second, no studies reported prevalence of eligibility for tezepelumab, the most recently approved biologic, which has the least restrictive licensing criteria with no biomarker requirements. Its inclusion would likely increase global prevalence estimates, meaning our results may underestimate the true proportion of biologic-eligible patients. Third, several studies in our review were single-centre cohorts, often from specialist clinics, which introduces selection bias and may limit generalizability. Future research should aim to include larger, multi-centre, and nationally representative cohorts to provide more robust and generalizable prevalence estimates.

In conclusion, we provided the first comprehensive global estimate of biologic eligibility among patients with ATS/ERS-defined severe asthma and highlighted differences in eligibility across individual biologics. The wide variation in prevalence between countries underscores the unequal access to biologics worldwide, even among well-resourced healthcare systems. Our findings point to 2 key areas for future research. First, larger studies in primary care populations, which better reflect the general asthma population, are needed to more accurately determine the prevalence of biologic-eligible patients and support healthcare planning. Second, research is urgently needed to address the large proportion of severe asthma patients who remain ineligible for any current biologic therapy. While tezepelumab, which was not included in our review, may partially fill this gap, it is unlikely to fully address the unmet needs of almost half of those with severe asthma.

## Confirmation of unpublished work

This manuscript is original, has not been published previously, and is not under consideration for publication elsewhere.

## Ethics statement

This study is a systematic review of previously published literature and did not involve the collection of new patient data.

## Authors contributions

FY, JDS and CIB developed the research question and study objectives. FY and JDS designed the search strategy, eligibility criteria, conducted the database searches, data extraction, performed risk of bias and quality appraisal of included studies. MM and CIB reviewed the articles for adjudication. FY conducted prevalence calculations and prepared the first draft of the manuscript. All authors critically reviewed the manuscript and contributed to revisions.

## Declaration of Generative AI and AI-assisted technologies in the writing process

We acknowledge that ChatGPT (OpenAI) was used solely to assist with improving the clarity and readability of the manuscript text. ChatGPT was not used to compose the manuscript, generate scientific content, provide clinical recommendations, or create or modify any figures, images, or artwork.

## Funding

This research was funded by the 10.13039/501100013342NIHR Imperial Biomedical Research Centre (BRC). The views expressed are those of the authors and not necessarily those of the NIHR or the Depart of Health and Social care. Open access fee was paid from the Imperial College London Open Access Fund.

## Conflicts of interest

FY has received honoraria for attending meetings and speaker fees from AstraZeneca and GlaxoSmithKline, and is a member of the British Thoracic Society Asthma Advisory Group. JDS is an employee of Upstream Bio and the entirety of this work was completed during his full-time employment at Brigham and Women's Hospital. REP and MM have no conflicts of interest to declare in relation to this work. CIB has received research funding from NIHR, Asthma + Lung UK and AstraZeneca, outside the scope of this work.
