# Supplementary material for: Global prevalence of eligibility for biologic therapy in ATS/ERS-defined severe asthma: A systematic review
Source: World Allergy Organ J. 2025 Dec 11;18(12):101155. doi: 10.1016/j.waojou.2025.101155 (PMC12757517; doi:10.1016/j.waojou.2025.101155)
Supplement: Multimedia component 1 [file mmc1.docx]

# SUPPLEMENTARY DOCUMENT

# Title: Global prevalence of eligibility for biologic therapy in ATS/ERS-defined severe asthma: A Systematic Review

# Supplement E1. Exclusion criteria used for selecting studies for review.

| **Studies were excluded if they did not meet the primary study objective. Specifically, we excluded:** | |
| --- | --- |
| 1 | **Duplicate publications**reporting results from the**same patient population**as a study already included. |
| 2 | **Study protocols or design papers**without patient-level eligibility data. |
| 3 | **Studies involving patients already receiving biologic therapy**at the time of assessment, including **biologic switch studies.** |
| 4 | **Studies assessing exacerbations only,** without biomarker evaluation for biologic eligibility. |
| 5 | **Studies exclusively enrolling children** (<12 years). |

# Supplement E2. Eligibility criteria used in studies investigating the proportion of patients eligible for omalizumab.

| **Author and year** | **Country** | **% Eligible** | **Criteria** |  |
| --- | --- | --- | --- | --- |
| **Albers 2018** | Global  (6 countries*) | 7.4% (UK/France/Germany)  21.3% (AUS/CAN/USA) | ATS/ERS severe asthma plus depending on country: Either ≥1 or ≥2 exacerbations requiring oral corticosteroids, emergency admissions or hospitalisation in previous 12 months; AND IgE levels and weight as per licencing criteria, RAST positive; AND some countries required FEV1 ≤80%. |  |
| **Deng 2023** | China | 11.3% | ATS/ERS severe asthma; AND ≥2 exacerbations in past year; AND presence of sensitization on SPT or specific IgE AND body weight within the dosage range. |  |
| **Lee 2019** | Korea | 6.3% | ATS/ERS severe asthma; AND criteria according to Korea Food and Drug Administration (KFDA) |  |
| **Marques Mello 2021** | Brazil | 34.9% | ATS/ERS severe asthma; AND persistent, allergic asthma with inadequate control despite inhaled corticosteroid; AND ≥1 exacerbation; AND meets IgE and weight dosing table. |  |
| **Kanniess 2021** | Europe  (12 countries**) | Unavailable | ATS/ERS severe asthma; AND label criteria in Western Europe |  |
| **Lee 2018** | Australia | 43.5% | ATS/ERS severe asthma; AND allergic phenotype (total IgE ≥30kU.mL-1); AND sensitization to an aeroallergen from SPT or serum specific IgE. |  |
| **Akenroye 2020** | USA | 41% | ATS/ERS severe asthma; AND inhaled corticosteroid + LABA / LAMA for at least 1 year OR oral corticosteroids for at least 3 months; AND ≥1 exacerbation in past year requiring urgent care or emergency admission; AND FDA criteria for omalizumab (age, weight, IgE and perennial sensitivity) |  |
| **Menzella 2020** | Italy | 7.3% | ATS/ERS severe asthma; AND age ≥6 and ≤75; AND total IgE >75 ≤1500IU/L; AND ≥1 exacerbation requiring oral corticosteroids, emergency admission or hospitalisation in past 12 months; AND positive SPT or serum specific IgE for perennial allergen; AND FEV1 <80% pre-bronchodilator. |  |
| **Jeimy 2018** | Canada (Ontario) | 66% | ATS/ERS severe asthma; AND age ≥6; AND IgE ≥30 ≤700; AND positive perennial aeroallergen with SPT or serum specific IgE; AND moderate-severe symptoms despite inhaled corticosteroids. |  |

| * | The 6 countries included Australia, Canada, France, Germany, UK, USA |
| --- | --- |
| ** | The 12 countries included Bulgaria, Czechia, France, Germany, Greece, Hungary, Italy, Netherlands, Poland, Romania, Slovenia, Spain |

**Abbreviations:**

ATS, American Thoracic Society; AUS, Australia; CAN, Canada; ERS, European Respiratory Society; FDA, Food and Drug Agency; FEV1, Forced Expiratory Volume in 1 second; LABA, Long-acting beta-agonist; LAMA, Long-acting anti-muscarinic; SPT, Skin Prick Test.

# Supplement E3. Eligibility criteria used in studies investigating the proportion of patients eligible for mepolizumab, benralizumab and reslizumab.

| **Author and year** | **Country** | **Mepolizumab** | **Benralizumab** | **Reslizumab** | **Criteria** |
| --- | --- | --- | --- | --- | --- |
| **Albers 2018** | Global  (6 countries*) | 20.1% | . | 5.6% | ATS/ERS severe asthma.  Mepolizumab: ≥2 exacerbations requiring oral corticosteroid, emergency admissions or hospitalisation in previous 12 months; AND BEC ≥150 at study visit or ≥300 past 12 months.  Reslizumab: ≥1 exacerbations requiring oral corticosteroid, emergency admission or hospitalisation in previous 12 months; AND BEC ≥400 at study visit, AND ≥12% airway reversibility to short-acting beta-2-agonist. |
| **Deng 2023** | China | 19.3% (anti-IL5) | 19.3% (anti-IL5) | 19.3% (anti-IL5) | ATS/ERS severe asthma; AND ≥2 exacerbations in past year; AND BEC ≥300. |
| **Lee 2019** | Korea | 36.7% | . | . | ATS/ERS severe asthma; AND according to Korea Food and Drug Administration (KFDA) |
| **Comberiati 2019** | USA | 55.1% (BEC ≥150)  38.0% (BEC ≥300) | . | . | ATS/ERS severe asthma; AND Age ≥12 AND ≥2 exacerbations in 12 months AND BEC ≥150 or ≥300 at study visit. |
| **Marques Mello 2021** | Brazil | 35.5% | 18.6% | 16.9% | ATS/ERS severe asthma.  Mepolizumab: severe eosinophilic asthma in adult patients; AND ≥2 exacerbations in 12 months; AND BEC ≥150.  Benralizumab: severe eosinophilic asthma adult patients; AND ≥2 exacerbations in 12 months; AND BEC ≥300.  Reslizumab: severe eosinophilic asthma adult patients; AND ≥1 exacerbation; AND BEC ≥400. |
| **Kanniess 2021** | Europe  (12 countries**) | Not specified | Not specified | Not specified | ATS/ERS severe asthma; AND label criteria in Western Europe |
| **Lee 2018** | Australia | 31.9% (anti-IL5) | 31.9% (anti-IL5) | 31.9% (anti-IL5) | ATS/ERS severe asthma; AND BEC ≥300. |
| **Akenroye 2020** | USA | 66.9% | 32.3% | 19.5% | Severe asthma AND inhaled corticosteroid + LABA / LAMA for at least 1 year OR oral corticosteroid for at least 3 months; at least 1 exacerbation in past year requiring urgent care or emergency visit; FDA blood eosinophil count for anti-IL5s but manuscript did not specify limits used. |
| **Menzella 2020** | Italy | 19.7% | 19% | . | ATS/ERS severe asthma.  Mepolizumab: age ≥18; AND BEC ≥150 at screening or ≥300 within 12 months; AND ≥2 exacerbations requiring oral corticosteroids, emergency admissions or hospitalisation in 12 months.  Benralizumab: age ≥18 ≤75; AND BEC ≥300 in 12 months; AND ≥2 exacerbations requiring oral corticosteroids, emergency admissions or hospitalisation in 12 months. |
| **Jeimy 2018** | Canada (Ontario) | 78% | 53% | 41% | ATS/ERS severe asthma.  Mepolizumab: Age >18; AND BEC ≥150 or ≥300 in previous 12 months; AND inadequate control despite high dose inhaled corticosteroids plus additional controller.  Benralizumab: Age >18; AND BEC ≥300 and ≥2 exacerbations in previous 12 months OR BEC ≥150 and ≥1 exacerbations previous 12 months and maintenance oral corticosteroids; AND inadequate control despite high dose inhaled corticosteroid and additional controller.  Reslizumab: Age >18; AND BEC ≥400; AND inadequate control despite high dose inhaled corticosteroids and additional controller. |

| * | The 6 countries included Australia, Canada, France, Germany, UK, USA |
| --- | --- |
| ** | The 12 countries included Bulgaria, Czechia, France, Germany, Greece, Hungary, Italy, Netherlands, Poland, Romania, Slovenia, Spain |

**Abbreviations:**

ATS, American Thoracic Society; BEC, Blood Eosinophil Count; ERS, European Respiratory Society; FDA, Food and Drug Agency; LABA, Long-acting beta-agonist; LAMA, Long-acting anti-muscarinic.

# Supplement E4. Eligibility criteria used in studies investigating the proportion of patients eligible for dupilumab.

| **Author and year** | **Country** | **Dupilumab** | **Criteria** |
| --- | --- | --- | --- |
| **Deng 2023** | China | 36.7% | ATS/ERS severe asthma; AND ≥2 exacerbations in past year; AND BEC ≥150 or FeNO ≥25ppb. |
| **Kanniess 2021** | Europe (12 countries*) | Not specified | ATS/ERS severe asthma; AND label criteria in Western Europe |
| **Akenroye 2020** | USA | 75.1% | Severe asthma PLUS inhaled corticosteroid/LABA +/- LAMA for at least 1 year or oral corticosteroids for at least 3 months; ≥1 exacerbation in past year requiring urgent care or emergency admission; FDA criteria for BEC but limit was not specified in manuscript AND also needed on oral corticosteroids for at least 90 days |

| * | The 12 countries included Bulgaria, Czechia, France, Germany, Greece, Hungary, Italy, Netherlands, Poland, Romania, Slovenia, Spain |
| --- | --- |

**Abbreviations:**

ATS, American Thoracic Society; BEC, Blood Eosinophil Count; ERS, European Respiratory Society; FDA, Food and Drug Agency; LABA, Long-acting beta-agonist; LAMA, Long-acting anti-muscarinic.

|  | **≥1 Biologic** | | | **Omalizumab** | | | **Mepolizumab** | | | **Benralizumab** | | | **Reslizumab** | | | **Dupilumab** | | |
| --- | --- | --- | --- | --- | --- | --- | --- | --- | --- | --- | --- | --- | --- | --- | --- | --- | --- | --- |
|  | **%** | **n** | **total** | **%** | **n** | **total** | **%** | **n** | **total** | **%** | **n** | **total** | **%** | **n** | **total** | **%** | **n** | **total** |
| Albers 2018* | 0.29 | 148 | 502 | 0.14 | 70 | 502 | 0.20 | 101 | 502 | . | 0 | . | 0.06 | 28 | 502 | . | . | . |
| Deng | 0.39 | 174 | 452 | 0.11 | 51 | 452 | 0.19 | 87 | 452 | 0.19 | 87 | 452 | 0.19 | 87 | 452 | 0.37 | 166 | 452 |
| Lee 2019 | 0.40 | 321 | 809 | 0.06 | 51 | 809 | 0.20 | 163 | 809 | . | 0 | . | . | 0 | . | . | . | . |
| Comberiati 2019** | 0.38 | 60 | 157 | . | 0 | . | 0.38 | 60 | 157 | . | 0 | . | . | 0 | . | . | . | . |
| Marques Mello 2021 | 0.60 | 103 | 172 | 0.35 | 60 | 172 | 0.36 | 61 | 172 | 0.19 | 32 | 172 | 0.17 | 29 | 172 | . | . | . |
| Kanniess 2021*** | 0.62 | 636 | 1025 | . | 0 | . | . | 0 | . | . | 0 | . | . | 0 | . | . | . | . |
| Lee 2018 | 0.64 | 38 | 59 | 0.44 | 26 | 59 | 0.32 | 19 | 59 | 0.32 | 19 | 59 | 0.32 | 19 | 59 | . | . | . |
| Akenroye 2020 | 0.84 | 50 | 59 | 0.41 | 24 | 59 | 0.67 | 39 | 59 | 0.32 | 19 | 59 | 0.20 | 12 | 59 | 0.75 | 44 | 59 |
| Menzella 2020 | 0.91 | 125 | 137 | 0.07 | 10 | 137 | 0.20 | 27 | 137 | 0.19 | 26 | 137 | . | 0 | . | . | . | . |
| Jeimy 2018 | 0.91 | 117 | 128 | 0.66 | 84 | 128 | 0.78 | 100 | 128 | 0.53 | 68 | 128 | 0.41 | 52 | 128 | . | . | . |
| **Total number** |  | **1770** | **3500** |  | **377** | **2318** |  | **657** | **2475** |  | **251** | **1007** |  | **227** | **1372** |  | **210** | **511** |
| **% of total** |  | **51%** |  |  | **16%** |  |  | **27%** |  |  | **25%** |  |  | **17%** |  |  | **41%** |  |

# Supplement E5. Collated patient numbers and calculated percentages from all included severe asthma studies, showing the proportion of patients eligible for at least one biologic and for each individual biologic.

| * | Average of Group 1 and Group 2 applied, as the number of patients in each group was not reported. |
| --- | --- |
| ** | Study reported blood eosinophil count thresholds of ≥150 and ≥300 cells/µL; the ≥300 cells/µL value was used. |
| *** | Proportions eligible for individual biologics were not reported. |

# Supplement E6. Studies in non‑severe asthma populations (difficult‑to‑treat, mild‑to‑moderate, and undefined asthma): proportion of patients eligible for biologic therapy.

| **Author**  **Year** | **Study**  **design** | **Study population** | **Age**  **(years)** | **Country** | **Omalizumab** | **Mepolizumab** | **Benralizumab** | **Reslizumab** | **Dupilumab** | **Total patients (*n)*** | **Percentage of biologic eligible patients** |
| --- | --- | --- | --- | --- | --- | --- | --- | --- | --- | --- | --- |
| **Dhruvel 2022** | Cross-sectional | Asthma (adults) | 18+ | UK (London) | ✓ | ✓ | ✓ | ✓ | ✓ | 2473 | 1.4% |
| **Ilmarinen 2019*** | Cross-sectional | Asthma (adult onset) | 15+ | Finland (Seinäjoki) |  | ✓ | ✓ | ✓ |  | 203 | 2% |
| **Akenroye 2020**** | Cross-sectional | Asthma | 6+ | USA | ✓ | ✓ | ✓ | ✓ | ✓ | 1853 | 2.8% |
| **Buhl 2013***** | Cross-sectional | Moderate-to-severe allergic asthma | 18+ | Italy and Germany | ✓ |  |  |  |  | 771 | 12% |
| **Burton 2010** | Cross-sectional | Difficult-to-treat asthma | 18+ | UK (Leicester) | ✓ |  |  |  |  | 510 | 13.1% licencing  (5.3% NICE) |
| **Molfino 2020** | Cross-sectional | Asthma (adults) | 18+ | USA |  | ✓ | ✓ | ✓ |  | 774 | 19.7% |
| **Heaney 2012***** | Cross-sectional | Difficult-to-treat asthma | 18+ | UK | ✓ |  |  |  |  | 582 | 30.6% licensing  (13.9% NICE) |
| **Lee 2018** | Cross-sectional | Difficult-to-treat asthma | 18+ | Australia | ✓ | ✓ | ✓ | ✓ |  | 69 | 55% |

| * | Ilmarinen (2019) was reported as a 12‑year cohort study (257 patients at baseline, 203 at follow‑up). However, it is considered cross‑sectional, as eligibility and exacerbations were assessed only for the 12 months preceding the final follow‑up. |
| --- | --- |
| ** | Akenroye (2020) included patients aged ≥6 years; only adult data (≥18 years) are reported here. The original study reported overall results for ≥6 years and separate subgroups for <18 and ≥18 years. |
| *** | Buhl (2010) and Heaney (2012) reported prevalence according to both licensing criteria and UK National Institute for Health and Care Excellence (NICE) criteria. |

# Supplement E7. Studies in non‑severe asthma populations (difficult‑to‑treat, mild‑to‑moderate, or undefined asthma): proportion of patients eligible for each biologic.

| **Author and year** | **Omalizumab** | **Mepolizumab** | **Benralizumab** | **Reslizumab** | **Dupilumab** |
| --- | --- | --- | --- | --- | --- |
| **Dhruvel 2022*** | Not specified | 0.81% (anti-IL5) | 0.81% (anti-IL5) | 0.81% (anti-IL5) | 0.28% |
| **Ilmarinen 2019*** | . | 1% (anti-IL5) | 1% (anti-IL5) | 1% (anti-IL5) | . |
| **Akenroye 2020** | 1.4% | 2.1% | 1.1% | 0.7% | 2.5% |
| **Buhl 2013**** | 12.0% | . | . | . | . |
| **Burton 2010** | 13.1% Licensing  (5.3% NICE) | . | . | . | . |
| **Molfino 2020** | . | 19.7% (anti-IL5) | 19.7% (anti-IL5) | 19.7% (anti-IL5) | . |
| **Heaney 2012**** | 30.6% licencing  (13.9% NICE) | . | . | . | . |
| **Lee 2018*** | 43.5% | 31.9% (anti-IL5) | 31.9% (anti-IL5) | 31.9% (anti-IL5) | . |

| * | Dhruvel (2022), Ilmarinen (2019), Molfino (2020) and Lee (2018) reported the proportion of patients eligible for anti-IL5/IL5Ra therapies as a combine group, rather than for individual biologics. |
| --- | --- |
| ** | Buhl (2010) and Heaney (2012) reported prevalence according to both licensing criteria and UK National Institute for Health and Care Excellence (NICE) criteria. |

# Supplement E8. Studies in non‑severe asthma populations (difficult‑to‑treat, mild‑to‑moderate, or undefined asthma): characteristic of studies.

| **First Author** | **Publication year** | **Article Type** | **Journal or Conference** | **Data source** | **Study start date** | **Study end date** |
| --- | --- | --- | --- | --- | --- | --- |
| **Dhruvel H** | 2022 | Conference abstract | BTS Winter Meeting | Single centre, primary care network (PCN), EMIS web | 2021 | 2021 |
| **Ilmarinen P** | 2019 | Original article | JACI in practice | Single centre, Seinäjoki adult asthma study (SAAS) | 1999 | 2002 |
| **Buhl R** | 2013 | Original article | Respiratory Medicine | Retail and hospital sales data | Unavailable | Unavailable |
| **Burton** | 2010 | Conference abstract | BTS Winter Conference | Single centre clinic data | Unavailable | Unavailable |
| **Molfino** | 2020 | Conference abstract | ATS Congress | 24 pulmonary and allergy clinics in US (GSK funded) | 21/01/2019 | 29/04/2019 |
| **Heaney LG** | 2012 | Conference abstract | ERS Congress | British Thoracic Society (BTS) difficult asthma registry | Unavailable | Unavailable |

**Abbreviations:**

ATS, American Thoracic Society; BTS, British Thoracic Society; ERS, European Respiratory Society; JACI, Journal of Allergy and Clinical Immunology.

# Supplement E9. Studies in non‑severe asthma populations (difficult‑to‑treat, mild‑to‑moderate, or undefined asthma): eligibility criteria used in studies investigating the proportion of patients eligible for omalizumab.

| **Author and year** | **Study population** | **Country** | **Prevalence of omalizumab candidates** | **Criteria** |
| --- | --- | --- | --- | --- |
| **Dhruvel 2022** | Asthma (adult) | UK (London) | Not specified | NICE Criteria for anti-IgE: High-dose ICS/LABA; AND sensitised to perennial allergen; AND IgE in dosing range; AND ≥4 exacerbations. Authors used blood eosinophil count <150 as third line after anti-IL5s and anti-IL4a in the UK. |
| **Akenroye 2020** | Asthma | USA | 1.4% | Severe asthma; ICS + LABA / LAMA for at least 1 year OR oral corticosteroids for at least 3 months; AND ≥1 exacerbation in past year requiring urgent care or ED visit; AND FDA criteria for omalizumab (age, weight, IgE and perennial sensitivity) |
| **Buhl 2013** | Moderate-to-severe allergic asthma | Italy and Germany | 12.0% | High-dose ICS/LABA; AND FEV1 <80%; AND 2 or more symptoms (either day or night-time) per week; AND ≥2 exacerbations last 12 months |
| **Burton 2010** | Difficult-to-treat asthma | UK (Leicester) | 13.1% Licensing (5.3% NICE) | Licencing criteria: IgE 30-1500iu/ml; AND weight 20-150kg; AND FEV1 <80%; AND positive skin prick test or specific IgE to perennial aeroallergen; AND ongoing symptoms despite high-dose combination inhaler.  NICE criteria: licencing criteria PLUS ≥2 hospital admissions or 1 admission plus 2 ED attendances. |
| **Heaney 2012** | Difficult-to-treat asthma | UK | 30.6% licencing (13.9% NICE) | Licencing criteria: IgE 30-1500iu/ml; AND weight 20-150kg; AND FEV1 <80%; AND positive skin prick test or specific IgE to perennial aeroallergen; AND high-dose ICS/LABA.  NICE guideline: licencing criteria PLUS ≥2 hospital admissions or 1 admission plus 2 emergency admissions/GP visits. |
| **Lee 2018** | Difficult-to-treat asthma | Australia | 43.5% | ATS/ERS severe asthma; AND allergic phenotype (total IgE ≥30kU.mL-1); AND sensitization to an aeroallergen from SPT or serum specific IgE. |

**Abbreviations:**

ATS, American Thoracic Society; AUS, Australia; British Thoracic Society; ERS, European Respiratory Society; FDA, Food and Drug Agency; FEV1, Forced Expiratory Volume in 1 second; GP, General Practice; LABA, Long-acting beta-agonist; LAMA, Long-acting anti-muscarinic; NICE, The National Institute for Health and Care Excellence; SPT, Skin Prick Test.

#

# Supplement E10. Studies in non‑severe asthma populations (difficult‑to‑treat, mild‑to‑moderate, or undefined asthma): eligibility criteria used in studies investigating the proportion of patients eligible for mepolizumab, benralizumab and reslizumab.

| **Author and year** | **Study population** | **Country** | **Mepolizumab** | **Benralizumab** | **Reslizumab** | **Criteria** |
| --- | --- | --- | --- | --- | --- | --- |
| **Akenroye 2020** | Asthma | USA | 2.1% | 1.1% | 0.7% | Inhaled corticosteroids + LABA / LAMA for at least 1 year OR oral corticosteroids for at least 3 months; at least 1 exacerbation in past year requiring urgent care or emergency department visits; FDA blood eosinophil count for anti-IL5s but manuscript did not specify limits used |
| **Dhruvel 2022*** | Asthma (adult) | UK (London) | 0.81% (anti-IL5) | 0.81% (anti-IL5) | 0.81% (anti-IL5) | NICE criteria for anti-IL5/5Ra: High dose inhaled corticosteroids/LABA or LAMA, BEC ≥300 AND ≥4 exacerbations or BEC ≥400 AND ≥3 exacerbations. |
| **Ilmarinen 2019*** | Asthma (adult onset) | Finland (Seinäjoki) | 1% (anti-IL5) | 1% (anti-IL5) | 1% (anti-IL5) | Mod-high inhaled corticosteroids/LABA; AND ≥2 exacerbations in previous year; AND BEC ≥300 or FeNO ≥50ppb. |
| **Lee**  **2018*** | Difficult-to-treat asthma | Australia | 31.9% (anti-IL5) | 31.9% (anti-IL5) | 31.9% (anti-IL5) | ATS/ERS severe asthma; AND BEC ≥300. |
| **Molfino 2020*** | Asthma (adult) | USA | 19.7% (anti-IL5) | 19.7% (anti-IL5) | 19.7% (anti-IL5) | Medium to high dose inhaled corticosteroid plus LABA +/- LAMA; AND ≥2 exacerbations in 12 months; AND BEC ≥150 in past 12 months. |

| * | Dhruvel (2022), Ilmarinen (2019), Molfino (2020) and Lee (2018) reported the proportion of patients eligible for anti-IL5/IL5Ra therapies as a combine group, rather than for individual biologics. |
| --- | --- |

**Abbreviations:**

ATS, American Thoracic Society; BEC, Blood Eosinophil Count; ERS, European Respiratory Society; EU, Europe; FDA, Food and Drug Agency; GINA, Global Initiative for Asthma; LABA, Long-acting beta-agonist; LAMA, Long-acting anti-muscarinic; NICE, The National Institute for Health and Care Excellence.

# Supplement E11. Studies in non‑severe asthma populations (difficult‑to‑treat, mild‑to‑moderate, or undefined asthma): eligibility criteria used in studies investigating the proportion of patients eligible for dupilumab.

| **Author and year** | **Type of study population** | **Country** | **Dupilumab** | **Criteria** |
| --- | --- | --- | --- | --- |
| **Akenroye 2020** | Asthma | USA | 2.5% | Inhaled corticosteroids/LABA +/- LAMA for ≥1 year or oral corticosteroids for at least 3 months; ≥1 exacerbation in past year requiring urgent care or emergency department visit; FDA criteria for BEC but limit was not specified in manuscript AND also needed on oral steroids for at least 90 days |
| **Dhruvel 2022** | Asthma (adult) | UK (London) | 0.28% | NICE Criteria for anti-IL4R: High-dose inhaled corticosteroid/LABA or LAMA; AND BEC 150-300 AND ≥4 exacerbations. |

**Abbreviations:**

ATS, American Thoracic Society; BEC, Blood Eosinophil Count; ERS, European Respiratory Society; FDA, Food and Drug Agency; LABA, Long-acting beta-agonist; LAMA, Long-acting anti-muscarinic; NICE, The National Institute for Health and Care Excellence.
